# Genome sequences of two cyanobacteria strains isolated from hornworts

**DOI:** 10.1128/mra.01118-24

**Published:** 2025-03-28

**Authors:** Warren Shou Leong Ang, Meghan M. Blaszynski, Jun B. Cai, Lindsey Markowitz, Eve A. Maunders, Andreas Norlin, Haley R. Womack, Fay-Wei Li

**Affiliations:** 1 Boyce Thompson Institute, Ithaca, New York, USA; 2 Department of Plant Biology, University of Illinois Urbana-Champaign, Urbana, Illinois, USA; 3 Carl R. Woese Institute for Genomic Biology, University of Illinois Urbana-Champaign, Urbana, Illinois, USA; 4 Department of Integrative Biology, Oregon State University, Corvallis, Oregon, USA; 5 USDA-ARS Bee Research Laboratory, Beltsville, Maryland, USA; 6 Department of Biology, University of Maryland, College Park, Maryland, USA; 7 School of the Environment, University of Queensland, Brisbane, Queensland, Australia; 8 College of Marine Science, University of South Florida, St. Petersburg, Florida, USA; 9 College of Natural Sciences and Mathematics, University of Denver, Denver, Colorado, USA; 10 Plant Biology Section, Cornell University, Ithaca, New York, USA; University of Strathclyde, Glasgow, United Kingdom

**Keywords:** hornworts, cyanobacteria, symbiosis, diazotrophs, nitrogen fixation

## Abstract

We report two complete genome assemblies of symbiotic cyanobacteria isolated from the hornwort species *Notothylas orbicularis* and *Phaeoceros carolinianus*. These new datasets will facilitate future comparative genomic studies across symbiotic cyanobacteria.

## ANNOUNCEMENT

Hornworts have evolved symbiotic relationships with cyanobacteria to gain access to bioavailable nitrogen (1). Studying the interaction between hosts and cyanobionts is of particular interest for agricultural engineering. Despite the identification of many cyanobionts (2), few have their entire genome sequenced (3).

Here, we assembled genomes of two cyanobacteria strains isolated from hornworts—“C110” from *Notothylas orbicularis* and “C117” from *Phaeoceros carolinianus*, both were collected from upstate New York (GPS coordinates for C110: 42.451961, –76.454326, C117: 42.343843, –76.269182) (2, 3). Cyanobiont colonies were excised from thalli, sterilized with 5% bleach, and placed on BG11 plates with 6–25 µmol m^−2^ s^−1^ light intensity in 16/8 hour light/dark cycle at 22°C (3, 4). To obtain pure cultures, single colonies were restreaked at least twice. Cyanobacteria were then cultured in liquid BG11 media in the same conditions as before but with constant shaking. Cell density was measured by estimating chlorophyll-*a* concentration (5). Total chlorophyll-*a* mass of 250 µg (C110) and 65 µg (C117) were harvested by centrifugation. Genomic DNA was isolated using a Wizard HMW DNA Extraction Kit (Promega) and quantified using the Qubit 1X dsDNA High Sensitivity Kit on a Qubit 4 fluorometer (Invitrogen). A total of 200 ng of DNA was used for library preparation with the Rapid Sequencing Kit V14 (SQK-RAD114, Oxford Nanopore Technologies, UK) without any size selection. DNA fragment shearing was minimized with careful handling and loaded onto a MinION R10.4.1 Flow Cell (Oxford Nanopore Technologies, UK) to sequence for 24 hours.

Unless otherwise stated, default settings were used for all software processing. Raw data were basecalled using Dorado v0.7.3 in super accuracy mode with duplex basecalling (https://github.com/nanoporetech/dorado). Duplex and simplex reads were combined to generate a total of 879,153,448 bases from 160,803 reads (N50: 71,877 bp) and 1,784,282,371 bases from 201,384 reads (N50: 18,452 bp) for C110 and C117, respectively. Reads were filtered based on a minimum length cutoff of 1 k for C110 and 30 k for C117, and a minimum quality cutoff of Q6 for C110 and Q20 for C117. Genomes were assembled using Flye v2.9.2 (6), which circularized contigs when sufficient overlaps were identified.

The assembly from C110 yielded 11 circular contigs and 1 linear contig, totaling 8,366,001 bp (41.42% GC content). The largest circular contig was 7,174,855 bp, while others ranged from 32,854 bp to 331,577 bp. For C117, the assembly yielded four circular and two linear contigs, totaling 8,449,107 bp (39.7% GC content). The largest circular contig was 7,883,191 bp, while others ranged from 86,831 bp to 280,883 bp. Both genomes fall within the expected size range of a typical cyanobacteria. Completeness of both genomes was analyzed using Compleasm v0.2.6 (7) which scored 99.69% and 99.16% for C110 and C117, respectively. Genomes were annotated using National Center for Biotechnology Information (NCBI’s) PGAP v6.8 (https://github.com/ncbi/pgap). The 16S rRNA sequence of C110 showed 99.60% identity with *Nostoc sp*. ATCC 53789 (GenBank CP046703.1) when BLAST against GenBank’s core_nt database and average nucleotide identity (ANI) analysis using FastANI v1.33 (8) gave a score of 96.98%. The 16S rRNA sequence of C117 showed 97.24% identity with *Cylindrospermum sp*. NIES-4074 (GenBank AP018269.1), and FastANI scored 79.57%.

## Data Availability

The total raw nanopore reads were deposited under the BioProjects PRJNA1164287 (SRA accession SRR30853084) and PRJNA1164657 (SRA accession SRR30853069) for C110 and C117 respectively. The assemblies are also available at GenBank under the accessions GCA_043099725.1 and GCA_043099735.1 for C110 and C117 respectively.
